# Anti interferon-gamma (IFNγ) monoclonal antibody treatment in a patient carrying an *NLRC4* mutation and severe hemophagocytic lymphohistiocytosis

**DOI:** 10.1186/1546-0096-13-S1-O68

**Published:** 2015-09-28

**Authors:** C Bracaglia, A Gatto, M Pardeo, G Lapeyre, W Ferlin, R Nelson, C de Min, F De Benedetti

**Affiliations:** 1 Division of Rheumatology Ospedale Pediatrico Bambino Gesù, Department of Pediatric Medicine, Rome, Italy; 2 Novimmune SA, Plan-les-Ouates, Geneve, Switzerland

## Background

A growing body of evidence, in animals and humans, suggest that IFNγ plays a pathogenic role in primary HLH (pHLH) and in secondary HLH, including macrophage activation syndrome. A pilot study in pHLH with NI-0501, an anti-IFNγ monoclonal antibody, is ongoing. Mutations in *NLRC4* have been recently reported to cause recurrent MAS and an increased production of IL-18, a cytokine known to induce IFNγ.

## Objectives

To report safety and efficacy of NI-0501 in a patient, carrying a de novo *NLRC4* mutation and presenting with severe recalcitrant HLH.

## Results

A 4.5 month-old Caucasian child presented at 20 days of age with fever, rash hepatosplenomegaly, pancytopenia, hypofibrinogenemia, hypertriglyceridemia, marked ferritin and sCD25 increase. Severe liver insufficiency, followed by multiorgan failure, required ICU admission. HLH diagnosis was based on 6 HLH-2004 criteria. Analysis of genes involved in familial-HLH and functional tests (perforin expression, degranulation and cytotoxicity) were negative. Subsequent analysis of the *NLRC4* gene showed a *de novo* missense mutation (c.1010 C>A, encoding p.T337N), absent in his parents. Elevated serum levels of IL-18 and spontaneous IL-18 production were documented, confirming the relevance of the *NLRC4* mutation. Treatment with high-dose glucocorticoids and cyclosporine-A led to progressive improvement. Development of sepsis triggered an HLH reactivation with ICU admission. Treatment with etoposide and/or ATG was not considered because of active infections in an immunocompromised child. Measurement of IFNγ and of the IFNγ-inducible chemokines showed measurable serum levels of IFNγ and high serum levels of CXCL9 (5670 pg/ml) and CXCL10 (4400 pg/ml). Compassionate use treatment with NI-0501 was started on background dexamethasone (13.6 mg/m2) and cyclosporine-A. He received NI-0501 for 3 months, initially every 3 days and subsequently every 7 days according to NI-0501 pharmacokinetics. No infusion reaction was observed. HLH clinical and laboratory features progressively improved. Glucocorticoid tapering was rapidly initiated. After 3 months, the child is in excellent conditions; all HLH clinical and laboratory parameters have normalized. CRP occasionally increases. Serum IL-18 levels remain elevated. High circulating levels of IFNγ complexed with NI-0501, reflecting the high IFNγ production are detectable, but fully neutralized as shown by undetectable levels of IFNγ-inducible chemokines. He is receiving oral cyclosporine-A (6 mg/kg) and prednisone (0.3 mg/kg equivalent to 0.9 mg/m^2^ of dexamethasone).

## Conclusions

In a patient, carrying a de novo pathogenic *NLRC4* mutation and presenting with severe recalcitrant HLH, NI-0501 administration was well tolerated allowing control of all HLH features, while enabling glucocorticoid tapering. No safety concern emerged.

